# Single Amino Acid Substitution N659D in HIV-2 Envelope Glycoprotein (Env) Impairs Viral Release and Hampers BST-2 Antagonism

**DOI:** 10.3390/v8100285

**Published:** 2016-10-14

**Authors:** François E. Dufrasne, Catherine Lombard, Patrick Goubau, Jean Ruelle

**Affiliations:** 1 AIDS Reference Laboratory, Medical Microbiology Unit (MBLG), Institut de Recherche Expérimentale et Clinique (IREC), Université catholique de Louvain, Avenue Hippocrate 54 B1.54.05, B-1200 Brussels, Belgium; francois.dufrasne@uclouvain.be (F.E.D.); patrick.goubau@uclouvain.be (P.G.); 2 Pôle de Pédiatrie (PEDI), Institut de Recherche Expérimentale et Clinique (IREC), Université catholique de Louvain, Avenue Mounier 52 B1.52.03, B-1200 Brussels, Belgium; catherine.lombard@uclouvain.be

**Keywords:** HIV-2, BST-2, tetherin, gp36, envelope glycoprotein, Env, restriction factors, viral antagonist, FRET, CRISPR/Cas9 knockout

## Abstract

BST-2 or tetherin is a host cell restriction factor that prevents the budding of enveloped viruses at the cell surface, thus impairing the viral spread. Several countermeasures to evade this antiviral factor have been positively selected in retroviruses: the human immunodeficiency virus type 2 (HIV-2) relies on the envelope glycoprotein (Env) to overcome BST-2 restriction. The Env gp36 ectodomain seems involved in this anti-tetherin activity, however residues and regions interacting with BST-2 are not clearly defined. Among 32 HIV-2 ROD Env mutants tested, we demonstrated that the asparagine residue at position 659 located in the gp36 ectodomain is mandatory to exert the anti-tetherin function. Viral release assays in cell lines expressing BST-2 showed a loss of viral release ability for the HIV-2 N659D mutant virus compared to the HIV-2 wild type virus. In *bst-2* inactivated H9 cells, those differences were lost. Subtilisin treatment of infected cells demonstrated that the N659D mutant was more tethered at the cell surface. Förster resonance energy transfer (FRET) experiments confirmed a direct molecular link between Env and BST-2 and highlighted an inability of the mutant to bind BST-2. We also tested a virus presenting a truncation of 109 amino acids at the C-terminal part of Env, a cytoplasmic tail partial deletion that is spontaneously selected in vitro. Interestingly, viral release assays and FRET experiments indicated that a full Env cytoplasmic tail was essential in BST-2 antagonism. In HIV-2 infected cells, an efficient Env-mediated antagonism of BST-2 is operated through an intermolecular link involving the asparagine 659 residue as well as the C-terminal part of the cytoplasmic tail.

## 1. Introduction

Human immunodeficiency virus type 2 (HIV-2) belongs to the *Retroviridae* family and can cause acquired immune deficiency syndrome (AIDS) as HIV-1 does. Whereas the latter virus is ubiquitous, HIV-2 is mainly localized in West Africa and was isolated for the first time in 1986 [1,2,3]. The highest prevalence of HIV-2 occurs primarily in Guinea-Bissau and Senegal [4]. Both types of HIV arose from different interspecies transmissions of simian immunodeficiency viruses (SIVs) naturally infecting African primates [2,5]. In untreated individuals, HIV-2 plasma viral load is generally lower compared to HIV-1, resulting in lower transmission rates [6]. Importantly, in most of the HIV-2-positive individuals the disease does not progress to AIDS, although those who reach this disease state present clinical symptoms identical to HIV-1 infected patients [7,8,9,10]. In the HIV-2 infected group, many individuals appear to manage or control the infection and are therefore called long-term non-progressors [2,8,9,11]. A recent study explained, at least partly, the disparity of this evolution by showing the importance of host genetic factors in disease progression: two different individuals infected with a closely related strain of HIV-2 could be either viraemic or long-term non-progressors [12].

Some human protein families act as host cell restriction factors or cellular antiviral factors. Among those, APOBEC3G (apolipoprotein B mRNA-editing enzyme), a cytidine deaminase, introduces several lethal G to A substitutions during viral RNA retrotranscription in the cell cytoplasm [13]. TRIM5α (tripartite motif 5-alpha) disrupts the viral uncoating [14]. BST-2 (also called tetherin or CD317), which restricts the viral budding of enveloped viruses at the cell surface [15,16,17,18], is an interferon-inducible factor specifically expressed in plasmacytoid dendritic cells, plasma cells, mature B and CD4^+^ T cells [15,19,20,21,22]. Consequently, BST-2 promotes endocytosis of the attached virions by recruiting the clathrin adaptor protein AP-2. Viral particles are then susceptible to internalization into endosomal compartments and undergo subsequent degradation [17,18,23,24,25]. Structurally BST-2 is a type II integral membrane protein, with the N-terminus inside the cell cytoplasm, a single membrane spanning domain, and a C-terminus modified by the addition of an unusual GPI (glycosylphosphatidylinositol) anchor [26,27,28,29,30].

Genetic analyses have demonstrated that BST-2 was under high positive selective pressure during mammalian evolution [31,32,33,34]. As BST-2 is a potent antiviral factor, mechanisms against tetherin activity were selected in the course of evolution in viruses. In HIV-1, the viral antagonist is the accessory protein Vpu (viral protein unique) [15,16,24,35] which interacts physically with BST-2 through its transmembrane domain and internalizes BST-2 via an ubiquitination mediated by β-transducin repeat-containing protein 2 (β-TrCP) [36,37,38,39,40,41,42]. Subsequently, BST-2 is sequestered in the intracellular compartments leading to a downregulation at the cell surface facilitating viral release [21,24,43,44]. Although most of SIVs use Nef (negative regulatory factor) as tetherin antagonist [35,43,45,46,47], HIV-2 relies on its envelope glycoprotein (Env) [48]. The HIV-2 *env* gene encodes two envelope glycoproteins from a proteinic precursor, cleaved into an external protein (HIV-2 gp105) and a transmembrane protein (HIV-2 gp36, or gpTM) [48,49]. The interaction between the viral protein and BST-2 at the cell surface leads to the endocytosis of the complex. However, Env does not promote degradation of BST-2 through the proteasome pathway as Vpu does [36,44]. Experiments with chimeric viruses showed that the Env regions required to antagonize BST-2 lie mostly in the HIV-2 gp36 ectodomain. However, amino acid residues involved in this antagonistic role are not clearly defined, except for the endocytosis motif (GYRPV) in the Env cytoplasmic tail (CT) that allows the internalization of the Env-BST-2 protein complexes [24,27,43,48]. A recent study [50] conducted with the HIV-2 ROD14 strain demonstrated that the residues K422 and A598 in the ectodomain of the gp are mandatory for BST-2 antagonism. Nevertheless this strain is poorly released from some cell types as compared to the other HIV-2 ROD strains [51] and ROD14 Env differs from the ROD reference strain by several mutations including seven amino acid substitutions and a large deletion in the CT [52].

In the present study, we mapped the residues involved in the binding ability and the antagonism of BST-2 by site-directed mutagenesis in the gp36 ectodomain coding region of a functional HIV-2 ROD molecular clone. This clone encodes an Env protein with a full cytoplasmic tail (158 amino acids [53]) and does not harbor the I312T or V536L substitutions observed in the ROD10 molecular clone [1,52,54]. In the present study we demonstrated the importance of the asparagine residue at position 659 in the HIV-2 gp36 ectodomain for the anti-tetherin function.

The cytoplasmic tail of the envelope protein could be required for effective BST-2 antagonism, because some regions interact with clathrin adaptors and are involved in the protein localization in the cell. Furthermore, the hypothesis of a full cytoplasmic tail required for anti-tetherin function is supported by a recent study reporting that a Nef-deleted SIVmac virus could reacquire functional BST-2 antagonism by introducing some compensatory changes in the Env cytoplasmic tail [27,55,56,57,58]. Therefore, we also tested here a virus presenting a C-terminal 109 amino acid truncation in the CT that is spontaneously selected in vitro when HIV-2 reference strains are cultured [49].

## 2. Materials and Methods

### 2.1. Cell Lines

HEK293T and H9 cells were obtained from the National Institutes of Health (NIH) AIDS Research and Reference Reagent Program (NIH ARRRP). HEK293T cells were maintained in Dulbecco’s modified Eagles’s medium (DMEM) (Thermo Fisher Scientific, Waltham, MA, USA) supplemented with 10% fetal bovine serum (FBS, HyClone™ FetalClone™ from GE Healthcare Life Sciences, Fairfield, CT, USA) and 1% gentamicin (Thermo Fisher Scientific). H9 cells were maintained in RPMI-1640 (Thermo Fisher Scientific) supplemented with 10% FBS and 1% gentamicin. Jurkat cells (clone E6-1) were obtained through the ATCC organization (Manassas, VA, USA) and maintained in RPMI-1640 GlutaMAX™ media (Thermo Fisher Scientific). All cells lines were tested for mycoplasma contamination before use.

### 2.2. Plasmids, Cloning and Site-Directed Mutagenesis

The pKP59 HIV-2 ROD proviral expression plasmid was obtained from Dr. Isabel Barahona (Instituto Superior de Ciências da Saúde-Sul, Portugal) [53]. This infectious clone encodes the whole genome of HIV-2 ROD reference strain (GenBank: M15390.1) and an envelope glycoprotein displaying a full cytoplasmic tail (158 amino acids). To generate all HIV-2 Env mutants tested in this study, site-directed mutagenesis was performed in order to introduce substitutions at the sites of interest in the pKP59-ROD infectious clone using the QuickChange II kit (Agilent Technologies, Santa Clara, CA, USA) and sequences were verified by nucleotide sequencing. HIV-2 envelope glycoprotein and *nef* sequences were amplified from the pKP59-ROD plasmid (primers *env*: 5′-ATGATGAATCAGCTGCTTATTGCC-3′; 5′-TCACAGGAGGGCGATTTCTGCTCC-3′ and primers *nef*: 5′-ATGGGTGCGAGTGGATCCAAG-3′; 5′-TTAACTAAATGGTATTCCTCTTGC-3′). Human *bst-2* sequence was amplified using cDNA obtained by reverse transcription of H9 cells mRNAs using the Transcriptor First Strand cDNA Synthesis Kit from Roche (Mannheim, Germany) (5′-ATGGCATCTACTTCGTATGAC-3′; 5′-TTACTGCAGCAGAGCGCTGAG-3′. NCBI Reference Sequence: NM_004335.3).

To customize and process the flow cytometry-based Förster resonance energy transfer (FRET) assay, pcDNA3-Clover (donor fluorochrome) and pcDNA3-mRuby2 (acceptor fluorochrome) expression plasmids were used. Both these FRET-adapted plasmids were a gift from Michael Lin (Departments of Pediatrics and Bioengineering, Stanford University, USA; Addgene plasmid # 40259, # 40260, respectively; [59]). The pcDNA3.1-Clover-mRuby2 was donated by Kurt Beam (Department of Physiology & Biophysics, University of Colorado, USA; Addgene plasmid # 49089) and served as a positive control. In order to fuse the Clover fluorochrome at the N-terminal part of protein, the tetherin sequence was amplified with primers including the restriction sites *Bsr*GI and *Apa*I (5′-TGTACAAGATGGCATCTACTTCGTATGAC-3′; 5′-GGCCCTTACTGCAGCAGAGCGCTGAG-3′). *Bsr*GI digestion of pcDNA-Clover removed the initial stop codon of the fluorochrome coding sequence, thereby enabling the correct expression of the Clover-BST-2 fused protein. Since the membrane localization signal of BST-2 is determined by both the anchor signal located in the transmembrane domain and the GPI-anchor, addressing of the BST-2 fusion protein is not disrupted in those conditions [26,28].

HIV-2 Env was fused with the mRuby2 fluorochrome on its C-terminal part. Env sequences from HIV-2 wild type (WT), Env 1–749 and Env N659D were amplified with primers including the restriction sites *Hind*III and *Nhe*I (for env WT and env N659D: 5′-AAGCTTATGATGAATCAGCTGCTTATTGCC-3′; 5′-GCTAGCCAGGAGGGCGATTTCTG-3′, and for env 1–749: 5′-GCTAGCGGGCCAGTATCTGTCTCCAC-3′ reverse primer) by taking care to remove the stop codon in the *env* sequence in order to enable the correct expression of the fused protein. For cloning HIV-2 *nef* sequence in pcDNA3-mRuby2, an analogous protocol was used, including *Hind*III-*Nhe*I sites. HEK293T cells (5 × 10^5^) were co-transfected with the following combination of constructed expression plasmids: 500 ng of plasmid expressing BST-2-Clover fusion protein and 1 μg of plasmid expressing Env-mRuby2 (or Env 1–749-mRuby2 or Env N659D-mRuby2) fusion proteins. A configured BD FACSAria™ III Cell Sorter (BD Biosciences, San Jose, CA, USA) was used to assess the binding capacity of HIV-2 Env WT and Env mutants proteins to BST-2 based on the FRET principle (a complete analysis of the cytometry platform configuration is detailed in the results section).

To perform *bst-2* knockout, a pL-CRISPR.EFS.GFP plasmid encoding *Streptococcus pyogenes* CRISPR associated protein 9 (SpCas9) and green fluorescent protein (GFP), a gift from Benjamin Ebert (Harvard Medical School, Boston, USA; Addgene plasmid # 57818, [60]), was used to transduce single guide RNA (sgRNA) targeting *bst-2* gene. Sequence fidelity for all constructed plasmids was verified by sequencing.

### 2.3. Cell Infections and Viral Release Quantification

The HIV-2 Env mutants selected using the comparison of the HIV-1 (HXB2, MN, BRU/LAI and BH10 strains) and HIV-2 (ROD, BEN, EHO and D205 strains) gp41/gp36 amino acid sequences were generated using site-directed mutagenesis from the pKP59-ROD infectious clone. HEK293T cells were transfected with these clones harboring mutations in Env. Briefly, 3 × 10^5^ cells were harvested and seeded in a 24-well plate 24 h before transfection. One microgram of pKP59-ROD was used to transfect cells with TurboFect Transfection Reagents according to the manufacturer’s instructions (Thermo Fisher Scientific). Three days post-transfection, supernatant was collected and centrifuged 10 min at 800 × *g*. Then, 5 × 10^5^ H9 cells were infected with 250 μL of infectious supernatant. Infected cells were washed three times with phosphate-buffered saline (PBS) (Thermo Fisher Scientific) after four hours of infection and then seeded in fresh RPMI-1640 media supplemented with 10% FBS and 1% gentamicin. Three days post-infection, the cell-free supernatant was filtered (0.45 μm) in order to quantify the viral particles in the cell culture medium and to establish virus stocks. For virus quantification, viral RNA was extracted with the MagNA Pure Compact Nucleic Acid Isolation Kit (Roche) and eluted in 50 μL. Three microliters of RNA served as a template for the reverse transcription (RT) reaction (Transcriptor First Strand cDNA Synthesis Kit from Roche). Three microliters of cDNA were added to a FastStart Essential DNA Green Master (Roche, Mannheim, Germany) containing primers specific to HIV-2 long terminal repeat (LTR; primers JR72 and JR73 [61]). Quantitative RT-PCR (RT-qPCR) was performed on a LightCycler^®^ 96 platform from Roche and absolute quantification (based on in vitro transcribed RNA calibrators [62]) was performed with the LightCycler^®^ 96 software.

For the viral release assays, a multiplicity of infection (MOI) of 2 was chosen to infect H9 and Jurkat cells (3 × 10^5^ cells infected with 6 × 10^5^ viral particles). Viral release was quantified by RT-qPCR at two, three and six days post-infection for the HIV-2 Env mutants tested in comparison to the HIV-2 WT virus. A negative control (supernatant from uninfected H9 or Jurkat cells) was tested in every viral quantification experiment. 

### 2.4. Subtilisin Treatment

Three days post-infection, the infected H9 cells were centrifuged (300 × *g* for 5 min). While the viral RNA present in the supernatant was stored for quantification, cell pellets were gently washed once with PBS, resuspended in 30 μL of subtilisin buffer (50 mM Tris-HCl pH 8, 150 mM NaCl, 5 mM CaCl_2_ in PBS 1X; concentration: 1 mg of subtilisin in 1 mL of buffer) and placed at 37 °C for 15 min. After incubation, 170 μL of phenylmethylsulfonyl fluoride (PMSF) (stock solution: 200 mM in 50 mL of culture medium) were added to the reaction to stop proteolysis, preserve cellular integrity and inactivate subtilisin protease (purchased from Sigma-Aldrich, St. Louis, MO, USA). Cells were centrifuged again and supernatant was collected to quantify the number of viral particles tethered at the cell surface. Two negative controls were tested for viral quantification: a medium from uninfected H9 cells treated with subtilisin, and a medium from infected H9 cells not treated with subtilisin.

### 2.5. CRISPR/Cas9 Knockout

The pL-CRISPR.EFS.GFP plasmid produced *bst-2* gene expression knockout in H9 cells. *Bsm*BI (*Esp3*I isoschizomer, Thermo Fisher Scientific) restriction sites were used to clone the sgRNA sequence (5′-CAGCGAGGCCTGCCGGGACGGCCTTCG-3′) downstream of a hU6 promoter in the pL-CRISPR.EFS.GFP plasmid. Its transcription products target the first exon of *bst-2* and allow Cas9 to produce a double-strand break in *bst-2*. Pseudotyped viruses were created by transfecting lentivectors in HEK293T cells: 750 ng of pL-CRISPR.EFS.GFP (encoding sgRNA of interest), 500 ng of psPAX2 (psPAX2 was a gift from Didier Trono (EPFL, Lausanne, Switzerland; Addgene plasmid # 12260) and 300 ng of pMD2.G encoding vesicular stomatitis virus envelope protein (VSV-G; Addgene plasmid # 12259). At 24 h after transfection, supernatant was collected, centrifuged to discard cells (or debris) and stored at 4 °C. Fresh cell culture medium was added to the virus-producing HEK293T cells for an additional 24 h. The pseudovirus-containing supernatants were pooled and used to infect H9 cells. After several passages, infected cells were sorted using a BD FACSAria™ III Cell Sorter (BD Biosciences, San Jose, CA, USA) according to GFP emission since Cas9 protein was fused to GFP. This transduction remained stable for approximately one month. Furthermore, Western blot was performed to confirm *bst-2* knockout. The viral release assay performed with these *bst-2* ^−^/^−^ H9 cells was the same as previously described. As a negative control, pL-CRIPSR.EFS.GFP encoding the initial sequence between *Bsm*BI restriction sites, which recognized the human cyclic adenosine monophosphate (cAMP) and cAMP-inhibited cyclic guanosine monophosphate (cGMP) 3′,5′-cyclic phosphodiesterase 10A intron sequence, was also used in this experiment.

### 2.6. Statistics and Sofware

Graphs and statistical analyses (Kruskal–Wallis one-way analysis of variance followed by Dunn’s multiple comparisons test) were performed using the GraphPad™ Prism 7.0 (GraphPad Software, La Jolla, CA, USA). A *p*-value ≤ 0.05 was considered to be statistically significant.

UniProt™ alignment tool allowed the comparison of the HIV-1 and HIV-2 gp41/36 sequences.

To analyze the fluorescence-activated cell sorting (FACS)-based FRET assay, FlowJo™ Data Analysis Software (version 10.1, Ashland, OR, USA) was used to generate multiple graphs.

## 3. Results

### 3.1. Residue N659 in the HIV-2 ROD Env Protein Is Involved in the Efficiency of Viral Release

**Figure 1:**
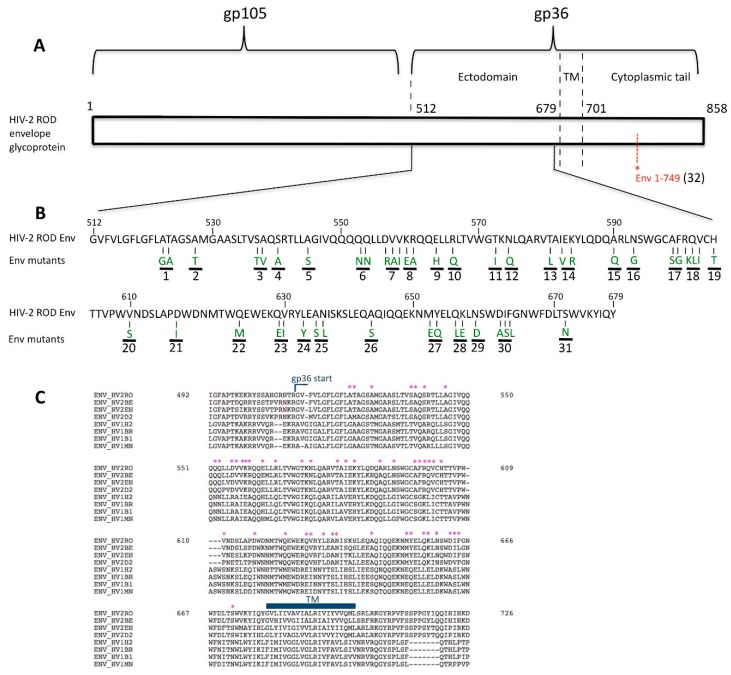
Schematic representation of human immunodeficiency virus type 2 (HIV-2) ROD envelope glycoprotein (Env). (**A**) The functional HIV-2 ROD Env protein is composed of an external domain (gp105) and a transmembrane domain (gp36). Three regions comprise gp36: the ectodomain, the membrane spanning domain and the cytoplasmic tail (158 amino acids). To generate a mutant virus with a truncated cytoplasmic tail (Env 1–749), the tryptophan residue (TGG) at the *env* position 749 was replaced with a stop codon (TAA) using site-directed mutagenesis. This position is indicated in red in this figure (TM: transmembrane domain); (**B**) Amino acids 512 to 679 composing the HIV-2 ROD gp36 ectodomain are shown in this panel. Based on a comparison of the HIV-1 and HIV-2 gp41/36 amino acid sequences, 47 positions were selected (Env mutants). All the selected amino acids mutations are highlighted in green, each corresponding to the related HIV-1 Env residues used to generate substitutions by side-directed mutagenesis of the pKP59 HIV-2 ROD plasmid. The 32 HIV-2 Env mutants generated in this study are numbered and highlighted with the black lines in this figure; (**C**) Alignment and comparison of the HIV-1 and HIV-2 gp41/36 amino acid sequences. Selected amino acids are pointed with the symbol * (Env_HV2RO: HIV-2 ROD strain; Env_HV2BE: HIV-2 BEN strain; Env_HV2EH: HIV-2 EHO strain; Env_HV2D2: HIV-2 D205 strain; Env_HV1H2: HIV-1 HXB2 strain; Env_HV1BR: HIV-1 BRU/LAI strain; Env_HV1B1: HIV-1 BH10 strain and Env_HV1MN: HIV-1 MN strain).

Since HIV-1 Env does not antagonize BST-2 [27], we selected 47 potential amino acids in HIV-2 Env by a comparison of the HIV-1 and HIV-2 gp41/36 amino acid sequences. Each conserved amino acid in HIV-2 reference strains but differing from HIV-1 reference strains HXB2, BRU/LAI, MN and BH10 was substituted with the corresponding HIV-1 residue. We used site-directed mutagenesis to introduce mutations at the sites of interest in a pKP59 (HIV-2 ROD) infectious clone and generated 32 different HIV-2 Env mutants (Figure 1).

**Figure 2:**
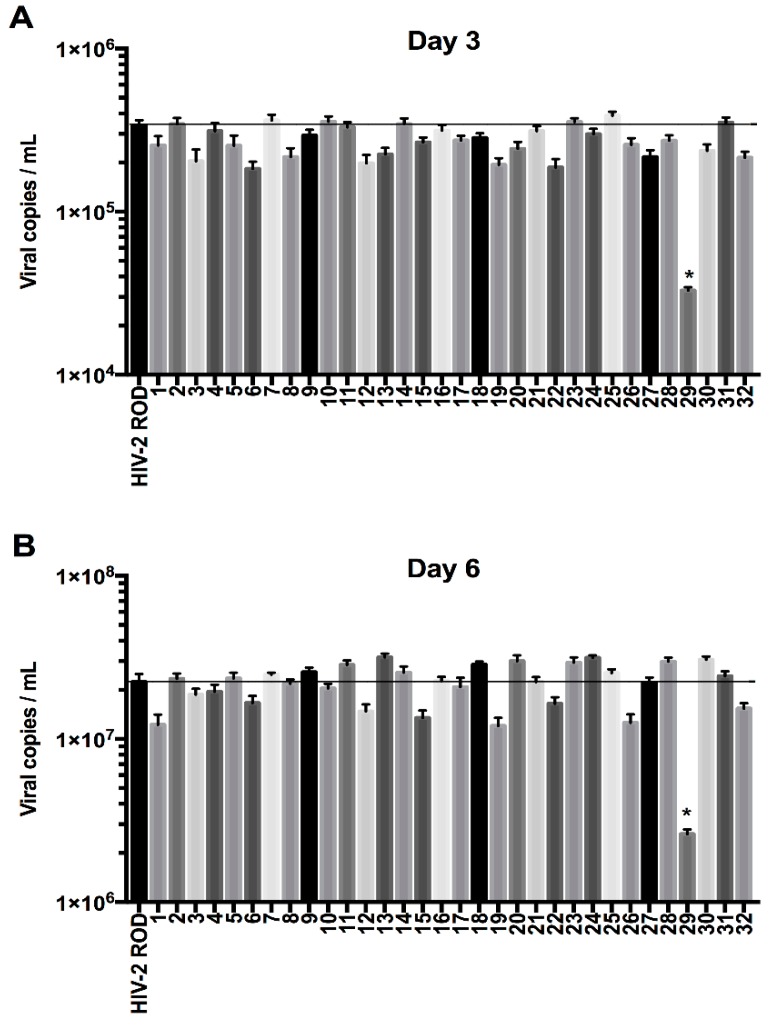
Absolute quantification of viral release ability among thirty-two HIV-2 Env mutants. The different clones were generated by site-directed mutagenesis and transfected in HEK293T cells. The viral particles were then used to infect H9 cells, a BST-2 producing cell line. Quantification was performed at three (**A**) and six (**B**) days post-infection (*n* = 3 independent experiments). The mutant numbers 1 to 32 are the same as highlighted substitutions in Figure 1B. The HIV-2 Env N659D mutant (mutant 29) showed a lower viral release compared to the HIV-2 Env WT. Error bars indicate mean ± standard deviation (SD) and statistical tests give statistical significance with a *p* < 0.05 (*).

The first objective was to find a mutant virus unable to antagonize BST-2, presenting thus a significant lack of release from infected cells since BST-2 inhibits the viral budding step in the viral cycle. HEK293T cells, which do not express BST-2, were transfected with the clones produced by mutagenesis and the released viral particles were collected in order to infect H9 cells, a cell line expressing BST-2 (MOI = 2). A RT-qPCR was performed to quantify viral particle release in the H9 cell culture medium. Since the HIV-2 Env cytoplasmic tail could be important in the Env-mediated anti-tetherin function as previously suggested [55,57,63], in this experiment we also tested the HIV-2 Env 1–749 mutant (mutant 32, Figure 1 and Figure 2) virus that expressed a truncated cytoplasmic tail (having a 49 amino acid CT length), in order to determine the impact of this size variability on the viral release ability and on the anti-tetherin activity.

In addition, we also tested a mutant carrying a similar mutation to HIV-2 Env ROD14 (substitution of the Env A598 residue) that was crucial for BST-2 antagonism in a recent study [50]. Intriguingly, in our hands this point mutation (mutant 17, Figure 1 and Figure 2) did apparently not cause a lack of viral release from H9 cells compared to HIV-2 Env WT in the context of the present study.

**Figure 3:**
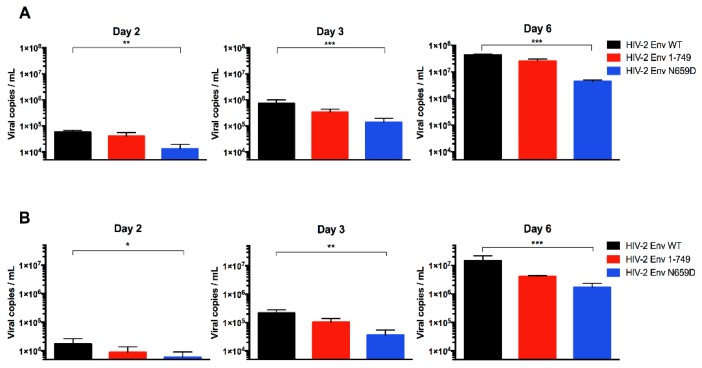
Absolute quantification of the HIV-2 Env wild-type (WT), Env 1–749 and Env N659D viral release capacity. (**A**) Quantification of the number of viral particles released from infected H9 cells in the supernatant at two, three and six days post-infection; (**B**) Quantification of the number of viral particles released from infected Jurkat cells in the supernatant at two, three and six days post-infection. Graphs present the number of viral copies in a logarithmic scale. Error bars indicate mean ± SD for *n* = 6 independent experiments. Statistical tests give statistical significance for HIV-2 Env WT and HIV-2 Env N659D with a *p* < 0.05 (*), *p* < 0.01 (**) or *p* < 0.001 (***).

Among the 32 HIV-2 Env mutants tested, the one harboring the N659D substitution showed a lower viral release from infected H9 and Jurkat cells compared to HIV-2 wild type virus at two, three and six days post-infection (Figure 3).

**Figure 4:**
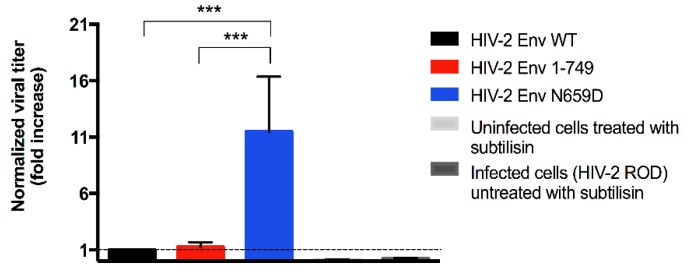
At three days post-infection, infected H9 cells were conserved and then treated with the bacterial protease subtilisin that released the tethered virions. Graph shows relative quantification of viral particles tethered at the cell surface. Data for the HIV-2 Env WT has been normalized to 1 (dashed line in the graph). Data for the others viruses has been compared to this normalized value and has been expressed as folds increase. Error bars indicate mean ± SD for *n* = 4 independent experiments. Statistical tests (one-way ANOVA followed by Tukey’s multiple comparisons test) give statistical significance with a *p* < 0.001 (***) both for Env WT and Env N659D viruses, and for Env 1–749 and Env N659D viruses. There is no statistical significance in titer between the Env WT and Env 1–749 viruses. Two negative controls were also tested: a medium from uninfected H9 cells treated with subtilisin, and a medium from infected H9 cells untreated with subtilisin.

In order to link the poorer release observed for the HIV-2 Env N659D virus to a viral inability to bud from the cell surface, we performed a subtilisin treatment of the infected H9 cells three days post-infection. As BST-2 inhibits the viral budding and retains the virions at the cell surface by embedding its GPI anchors into the budding virus membrane [21,29], subtilisin protease cleaves BST-2 peptides and allows the release of tethered virions. We performed RT-qPCR to quantify the number of tethered virions for H9 cells infected with the HIV-2 Env WT, HIV-2 Env 1–749 or HIV-2 Env N659D viruses. This experiment suggested a deficit of BST-2 antagonism for the N659D mutant compared to the Env WT and Env 1–749 viruses. Indeed, the HIV-2 N659D virus was eleven-fold more tethered at the cell surface (Figure 4).

### 3.2. Viral Release of the Env N659D Mutant Is Restored in BST-2-Depleted H9 Cells

In order to prove that the lack of viral release of the Env N659D mutant is linked to a deficit of BST-2 antagonism, we generated *bst-2* knockout in H9 cells using the CRISPR/Cas9 system. Pseudotyped viruses were used to transduce a sgRNA and the Cas9 endonuclease gene from *Streptococcus pyogenes* into H9 cells that naturally express BST-2. The single guide RNA is a synthetic RNA composed of a scaffold sequence mandatory for Cas9-binding and a 25 nucleotide sequence which targets the first exon of *bst-2* gene. Cas9 protein induced a double-strand break in the gene sequence and compelled the cells to introduce insertions or deletions (InDels). These InDels in the *bst-2* gene caused frameshift mutations within the open reading frame and ensured complete BST-2 knockout.

**Figure 5:**
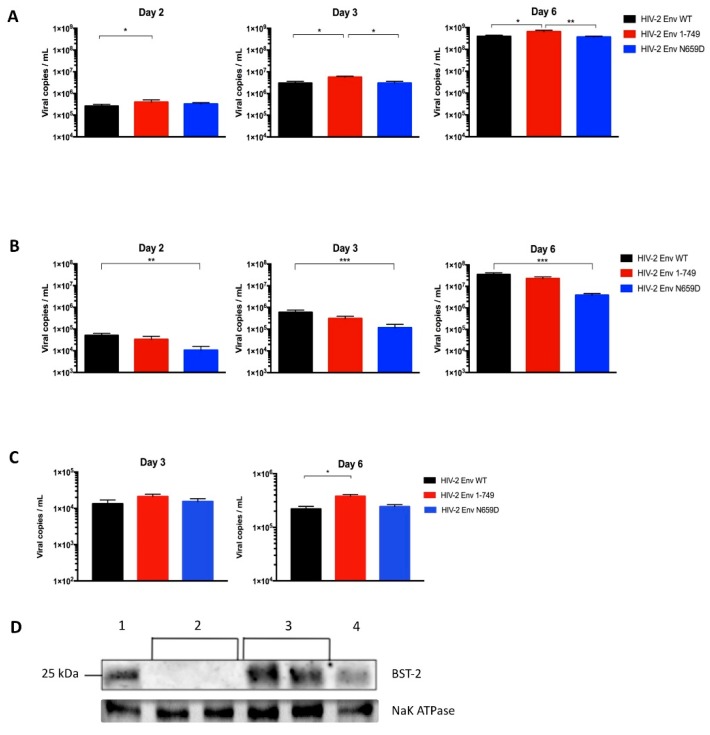
(**A**) Absolute quantification of the HIV-2 Env WT, Env 1–749 and Env N659D viral release capacity from BST-2-depleted H9 cells at two, three and six days post-infection; (**B**) Absolute quantification of viral particles released from H9 cells transduced with pseudotyped viruses that encoded Cas9 protein and a single guide RNA (sgRNA) sequence recognizing an intron sequence. The replicative capacity of the three viruses remained equivalent to the viral release assay using H9 cells that expressed BST-2 (Figure 3A); (**C**) Absolute quantification of viral release capacity from transfected HEK293T cells, which do not express BST-2, at three and six days post-transfection; (**D**) BST-2 expression in those cells was verified by immunoblotting proteins from H9 cells lysates (1), two different populations of BST-2-depleted H9 cells (2), two different populations of H9 cells transduced with pseudotyped viruses that encoded a sgRNA sequence recognizing an intron sequence (3) and Jurkat cells (4). Graphs present the number of viral copies in a logarithmic scale. Error bars indicate mean ± SD for *n* = 4 independent experiments (except for the panel **C**, *n* = 3). Statistical tests give statistical significance with a *p* < 0.05 (*), *p* < 0.01 (**) or *p* < 0.001 (***).

As expected, we found that particle release at two, three and six days post-infection of the Env N659D virus came back to a level comparable to the wild type (Figure 5A). These results confirmed that the N659 residue is involved in the Env-mediated anti-tetherin function in the HIV-2 ROD virus. Since BST-2 was not expressed in those H9 clones, the viral release efficiency was increased regardless of the three viruses tested when compared to the viral release observed in H9 cells that express BST-2 (Figure 3A). It is also interesting to note that the Env 1–749 virus, with a truncated cytoplasmic tail spontaneously selected after several passages during replication in vitro in some cell types [49], appeared to have a significant replicative advantage when produced in BST-2-depleted H9 cells.

As a control, we also tested our viruses in H9 cells transduced with pseudotyped viruses that encoded a sgRNA sequence recognizing the human cAMP and cAMP-inhibited cGMP 3′,5′-cyclic phosphodiesterase 10A intron sequence. BST-2 remained constitutively expressed in those cells and the replicative capacity was comparable to the viral release assay using untransduced H9 cells (Figure 5B). This assay represents a convincing control for ensuring that results obtained using BST-2-depleted cells were not dependent on or specific to the CRISPR/Cas9 knockout system. BST-2 expression in those cells was verified by immunoblotting proteins from H9 cells, from the different populations of BST-2-depleted H9 cells and from Jurkat cells lysates. A loading control was performed to conclude that BST-2 knockout was successful. Na^+^/K^+^-ATPase is also a membrane protein constitutively expressed in human cells (Figure 5D).

### 3.3. HIV-2 Env N659D Mutant Is Unable to Bind BST-2

We determined the binding capacity of this recombinant protein with BST-2 using a flow cytometry-based FRET assay. FRET is a robust and non-invasive approach to analyze protein interactions in living cells and is based on energy transfer between two compatibles fluorochromes. This transfer is efficient only if both fluorochromes are at a distance of less than 10 nm and the fluorescence emission intensity of the acceptor fluorochrome is enhanced [64].

**Figure 6:**
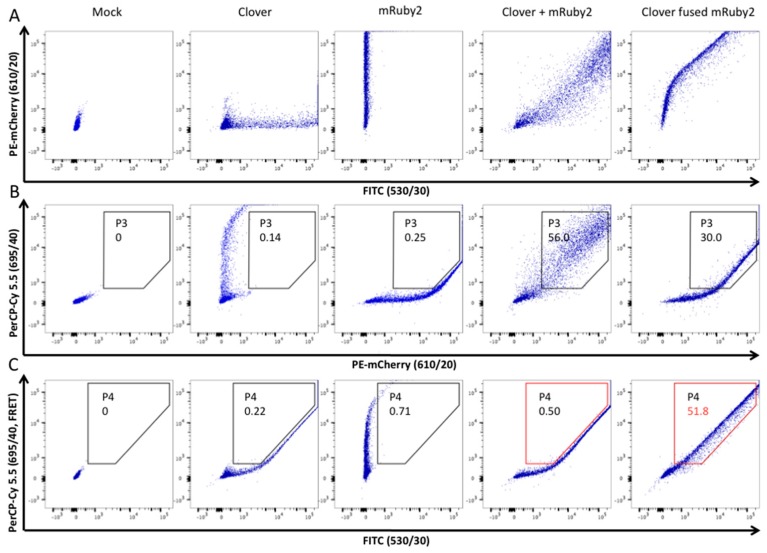
BD FACSAria™ III cell sorter configuration and experimental setup of Förster resonance energy transfer (FRET)-measurements. Firstly, gate P1 selecting living cells (according to forward and sideward scatter FSC-A/SSC-A) and gate P2 selecting cells in P1 (according to forward and sideward scatter FSC-W/SSC-W in order to exclude joined or grouped cells), have been applied (not shown in this figure). Secondly, HEK293T cells expressing Clover or mRuby2 individually, in combination or as a fusion protein, were analyzed with three different filters in order to construct analysis gates and to define the FRET-positive signal. Clover (green fluorescent protein variant) was excited with the laser 488 nm and the FITC filter was used to examine fluorescence emission. mRuby2 (red fluorescent protein variant) was excited with the laser 561 nm and the PE-mCherry filter was used (panel **A**). Importantly, mRuby2 and Clover showed some emission in the FRET-channel (PerCP-Cy 5.5 filter). Thus, a gate (P3) was constructed to exclude cells that emitted a false-positive signal in the FRET-channel (panel **B**). As described in this image, cells co-transfected with Clover and mRuby2 exerted an aleatory FRET signal that should also be excluded. Therefore, an analysis gate (P4, in red) was applied to determine the FRET-positive cells when the FRET-adapted filters were selected (PerCP-Cy 5.5 and FITC filters, panel **C**). This P4 gate excluded cells that were co-transfected with Clover and mRuby2 and thus are FRET-negative (0.5% of P2), while including cells that showed a FRET-positive signal (Clover fused mRuby2; 51.8% of P2). This gating strategy allowed for assessment the enhanced emission of the acceptor fluorochrome and therefore demonstrated the energy transfer between the two fluorochromes; namely the protein interactions.

Initially, FACS was not set up to assess FRET signal. To establish this experiment, we configured a BD FACSAria™ III cell sorter (BD Biosciences) as shown previously [65]. Two plasmids encoding each a FRET-adapted fluorochrome were used: pcDNA3-Clover and pcDNA3-mRuby2 encoding respectively Clover (a GFP variant) and mRuby2 (a red fluorescent protein (RFP) variant) [59]. As a positive control, we used a plasmid that expressed Clover fused to mRuby2 and an unmodified pcDNA3.1(+) as a negative control. Those four previous plasmids were transfected individually or in combination into HEK293T cells in order to select the cell sorter filters, exclude double false-positive and aleatory FRET signals, and finally to construct analysis gates to assess the specific FRET-measurements indicating relevant protein interactions (Figure 6).

**Figure 7:**
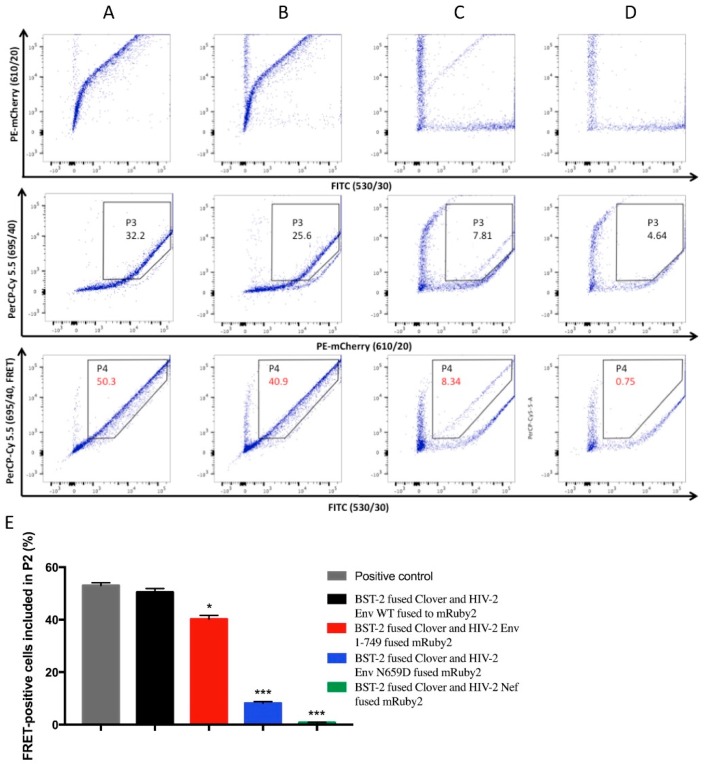
HEK293T cells co-expressing the fusion proteins in the following combinations were analyzed with the configured BD FACSAria™ III cell sorter. (**A**) BST-2 fused Clover and HIV-2 Env WT fused to mRuby2; (**B**) BST-2 fused Clover and HIV-2 Env 1–749 fused mRuby2; (**C**) BST-2 fused Clover and HIV-2 Env N659D fused mRuby2 and (**D**) BST-2 fused Clover and HIV-2 Nef fused mRuby2. Numbers indicated in gate P4 give the percentage of FRET-positive cells included in P2 according to the previous fluorescence-activated cell sorting (FACS) configuration for the different co-expression in living cells (Figure 6); (**E**) Bar diagram summarizing the FRET-positive signals from four independent experiments (*n* = 4). Statistical tests give a significance with a *p* < 0.05 (*), *p* < 0.01 (**) or *p* < 0.001 (***).

Once the cytometer was configured, we exploited this methodology in order to evaluate the capacity of the HIV-2 Env WT, Env 1–749 and Env N659D proteins to link with BST-2. We therefore constructed expression plasmids encoding the following fusion proteins: BST-2 fused to Clover on its N-terminal part and the three studied Env proteins each fused to mRuby2 on their C-terminal part. In this way, both FRET-adapted fluorochromes were localized in the cytoplasmic compartment of the living cells and thus allowed potential energy transfer between the fluorochromes. In addition, we constructed an expression plasmid that encoded HIV-2 Nef protein, since this myristoylated HIV-2 protein does not antagonize or interact with BST-2 [45,46,47,55]. These plasmids were co-transfected in combinations into HEK293T cells and the FRET-measurements were performed after thirty-six hours (Figure 7).

Firstly, this assay confirmed an effective intermolecular link between HIV-2 Env and human BST-2 proteins. Compared to the FRET signal exhibited by the positive control used to configure the FACS (Clover fused mRuby2, Figure 6C, plot 5), the FRET signal provided by cells co-expressing BST-2 and HIV-2 Env fusion proteins was comparable (50.3% of cells included in P2, Figure 7A); Secondly, a full cytoplasmic tail was required for the Env-mediated anti-tetherin function, as the FRET signal emanating from cells co-expressing BST-2 and HIV-2 Env 1–749 revealed a loss of binding capacity compared to HIV-2 Env WT (40.9% of cells in P2, Figure 7B). Conversely, the FRET signal was considerably diminished from cells co-expressing BST-2 and HIV-2 Env N659D, therefore illustrating a consistent lack of binding capacity (8.34%, Figure 7C). Thirdly, the absence of FRET signal (0.75%, Figure 7D) when BST-2 was co-expressed with HIV-2 Nef protein confirmed that these two proteins did not interact, as previously described in several studies [45,46,47,55].

This co-transfection was a robust negative control to evaluate our flow cytometry-based FRET assay and therefore allowed us to precisely determine protein interactions in living cells.

## 4. Discussion

BST-2 is part of the interferon (IFN)-dependent antiviral response pathway and acts as a potent host restriction factor [19,20,21,25]. Embedded into the cell membrane, mainly in cholesterol-rich domains where virions preferentially bud, this small protein blocks the viral release of enveloped viruses and therefore impairs viral spread [19,29,43]. Retroviruses evolved in turn several counteractions to evade this antiviral factor. Whereas HIV-1 relies on Vpu, HIV-2 uses its envelope glycoproteins to overcome BST-2 restriction and promote a downmodulation of the number of BST-2 molecules at the cell surface, facilitating the later viral budding from infected cells [44,48]. Env regions and residues involved in anti-tetherin activity are still less known and, to date, studies that aimed to define these regions have used particular Env proteins from the HIV-2 ROD10 or ROD14 molecular clones. These Env proteins displayed several specific mutations and both harbored a truncated cytoplasmic tail, which might alter structural conformation and functionalities of the envelope glycoprotein [50,51,52,54,66]. In contrast, replicating HIV-2 viruses from infected individuals encode a full cytoplasmic tail [49]. In this study, we pointed out a residue in the gp36 ectodomain essential for the antagonism of human BST-2.

We selected potential amino acids mutations by comparing the HIV-1 and HIV-2 gp41/36 sequences, since HIV-1 Env does not antagonize BST-2. Substitutions of HIV-2 Env residues with the corresponding HIV-1 amino acids allowed for preservation of functionalities and the tertiary structure of the envelope glycoprotein. We generated thirty-two Env mutant viruses and measured the impact of each mutation on their replicative capacity and ability to bud, allowing the identification of residues involved in the antagonism of BST-2. Our protocol was based on the quantification of HIV-2 viral particles released from human cell lines, which differs from a previous publication observing the stimulation of HIV-1 Gag-Pol virus-like particles (VLPs) release by HIV-2 Env variants [50]. Interestingly, we demonstrated that only a single substitution of the asparagine in position 659 (Env N659D) markedly impaired the viral release ability compared to the HIV-2 ROD wild type virus in two different T lymphocytes cell lines.

Subtilisin treatment revealed that the HIV-2 Env N659D virus was eleven-fold more tethered at the cell surface than HIV-2 Env WT or Env 1–749 viruses and thus presented an inability to be released from H9 cells. This experiment proved that the decrease of viral release was not due either to a lack of viral production or to a disruption of the Env addressing, as virions were actually localized at the cell surface. HIV-2 Env N659D virus is therefore unable to antagonize BST-2 and does not promote the endocytosis and sequestration of BST-2. Consequently, BST-2 remains localized at the cell surface and retains a large amount of budding viruses.

As a deficiency of viral release is not necessarily assigned to a lack of BST-2 antagonism, and as entry defaults could potentially influence viral titers in multiple rounds of infections, we performed a *bst-2* knockout in H9 cells using the CRISPR/Cas9 system. This key experiment indicated that the viral replication and release of the Env N659D virus were restored to a comparable level as with the wild type virus. These results also proved that the infectivity of the mutant virus was maintained, excluding an influence of the Env mutation on entry steps. Therefore, that asparagine residue in the gp36 ectodomain of the HIV-2 ROD virus is implicated in the Env-mediated anti-tetherin activity.

The N659 residue in Env might lie in the putative membrane-proximal external region (MPER). In HIV-1, this 22 amino acid peptide is involved in the incorporation of Env proteins into the membrane, allows membrane fusion, promotes viral infectivity and finally the MPER region is also an immunogenic peptide [67,68,69,70]. The N659D substitution could potentially have an impact on the HIV-2 predicted MPER functions. But when the asparagine residue was replaced with the equivalent HIV-1 residue, the viral assay using BST-2-depleted cells indicated that this mutant virus conserves its viral infectivity since viral release ability remained the same as with the HIV-2 Env WT virus. Moreover, it has been reported that only tryptophan residues in the MPER region are essential for membrane fusion and thus for viral infectivity [71].

Even though the HIV-2 Env region interacting with human BST-2 lies in the ectodomain, the cytoplasmic tail is also required for a correct antagonism: in this study we demonstrated that HIV-2 Env 1–749 presenting a truncated cytoplasmic tail partially lost its ability to bind BST-2. Interestingly, we previously showed that this mutant virus was spontaneously selected after several passages when cultured in some cell types [49], despite the fact that such a modified CT was not observed in clinical samples, suggesting that this shorter form is more vulnerable in vivo. This mutant with a truncated CT showed here a non-significant decline of its ability to be released from infected cells (H9 and Jurkat) compared to HIV-2 Env WT virus, while it was not more tethered at the cell surface as revealed by the subtilisin treatment. Conversely, that mutant had a significant replicative advantage when the replication occurred in BST-2-depleted H9 cells, suggesting that the spontaneous selection of this large C-terminal deletion occurs in cell lines that do not express large amounts of BST-2, such as MT-2 or MT-4 cells. The cytoplasmic tail is probably necessary in cells that constitutively express large amounts of BST-2. The argument that a full cytoplasmic tail is required for an adequate anti-tetherin function is supported by a recent study reporting that a Nef-deleted SIVmac virus could revert to a functional BST-2 antagonist by introducing some compensatory changes in its Env cytoplasmic tail [55]. In accordance with these insights, we demonstrated with a FRET assay the influence of the CT in the binding ability to BST-2, as cells co-expressing Env 1–749 and BST-2 showed a partial loss of protein interaction compared to cells co-expressing BST-2 and WT proteins. Moreover, our sensitive FRET experiments also confirmed the absence of interaction between HIV-2 Nef and BST-2 proteins. Although the experiments presented here all implied membrane localization of the Env variant proteins, further investigations would be needed to clarify the Env trafficking and addressing inside the cell, especially for the Env 1–749 proteins.

In summary, an efficient Env-mediated antagonism of BST-2 is operated through a direct intermolecular link, and an asparagine residue at the position 659 together with a normal full cytoplasmic tail are mandatory.

## 5. Conclusions

Among a series of 32 Env mutants generated by site-directed mutagenesis, we demonstrated the importance of the asparagine residue at position 659 in the HIV-2 gp36 ectodomain for the anti-tetherin function. Viral release ability in cell lines expressing BST-2 in comparison to *bst-2* knockout clones, subtilisin treatment of infected cells as well as FRET assays all point out the importance of this residue for antagonizing BST-2.

We also demonstrated the involvement of the Env cytoplasmic tail in this antagonistic role as viruses harboring a truncated cytoplasmic tail lost partially their function. Further investigations would be helpful to assess the envelope glycoprotein topology and structural conformation of those mutants, as well as the relevance of the various BST-2 antagonism abilities of HIV-2 strains in disease progression and non-progressing phenotypes.
